# The central clinical relevance of near-death experiences in acute care contexts: identification, prediction, and management

**DOI:** 10.3389/fpsyg.2025.1544438

**Published:** 2025-07-22

**Authors:** Pascal Michael, Pauline Fritz, Olivia Gosseries, Anne-Françoise Rousseau, Aurore Ancion, Alexandre Ghuysen, Charlotte Martial

**Affiliations:** ^1^Centre for Mental Health, School of Human Sciences, Old Royal Naval College, University of Greenwich, London, United Kingdom; ^2^Coma Science Group, GIGA-Consciousness, University of Liège, Liège, Belgium; ^3^Department of Neurology, NeuroRehab and Consciousness Clinic, University Hospital of Liège, Liège, Belgium; ^4^Department of Intensive Care, University Hospital of Liège and University of Liège, Liège, Belgium; ^5^Research Unit for a Life-Course Perspective on Health & Education (RUCHE), University of Liège, Liège, Belgium; ^6^Department of Emergency, University Hospital of Liège and University of Liège, Liège, Belgium; ^7^Department of Public Health, Medical Simulation Center, Liège University, Liege, Belgium

**Keywords:** near-death experience, emergency, intensive care, critical illness, memory

## Abstract

Near-death experiences (NDEs), a syndrome of experiences with mystical-type content classically arising in the context of life-threatening situations, are under-researched in terms of their relevance for acute medical care. We here discuss several reasons to raise the importance of conducting more comprehensive NDE research in emergency and critical care contexts, including but not limited to near-death experiencers’ awareness of surroundings and the need for patient support given NDEs’ profound psychological impacts, and we suggest incorporating the identification of NDEs into management plans. Exploring NDE incidence and their subsequent impact in acute settings may unveil a pathway toward favorable outcomes within clinical practice.

## Introduction

A strikingly large number of people claim to have had a near-death experience (NDE) with an incident rate that seems to be as high as 20% of cardiac arrest survivors (Schwaninger et al., 2002; van Lommel et al., 2001) and 15% of intensive care unit (ICU) survivors (Rousseau et al., 2023). Yet only very few prospective studies have investigated their incidence and potential risk factors in the classical contexts of their occurrence, as its original terminology indicates, i.e., in a state of near death (Schwaninger et al., 2002; van Lommel et al., 2001; Rousseau et al., 2023; Greyson, 2003a; Hou et al., 2013; Klemenc-Ketis et al., 2010; Mauduit et al., 2021; Parnia et al., 2023; Sabom, 1982; Sartori, 2008). In addition, the few existing prospective studies on NDE in critically ill patients were carried out only in homogeneous aetiologies, such as cardiac arrest or trauma survivors, with the exception of one prospective study that has explored the incidence of NDE in all aetiologies associated with ICU stay (Rousseau et al., 2023). As such, this article hopes to delineate why more dedicated research on and management of NDEs in such acute care contexts is necessary and would be invaluable for patient wellbeing and psychological outcomes.

## Enduring effects of NDEs

A more thoroughly studied aspect of the phenomenon of NDEs—although based instead on retrospective research—is its transformative impact, in terms of acute and prolonged consequences on psychological health, psychosocial and spiritual attitudes. These include (but are not limited to) significantly increased self-reported acceptance and empathy to others, connection to nature, reduced death anxiety and belief in materialism, as well as elevated sense of purpose, meaning, spirituality and gratitude (van Lommel et al., 2001; Groth-Marnat and Summers, 1998). Most of these appear objectively desirable in nature, and the majority of NDEs seem to be of a positive valence—even transcendently so—but even in these cases, they may bring their unique integrational challenges from attempts to reconcile, for instance, ontological belief shifts, which may also have inter-personal ramifications (Pratte, 2021). Equally, at least 14% of NDEs—which may be an underestimation due to response bias—have been retrospectively shown to be, sometimes deeply, distressing and have possibly significant implications for traumatic or emotional distress (Cassol et al., 2019). While still carefully acknowledging this, alongside the finding that those in a life-threatening situation who also report NDEs are more likely to report intrusive thoughts compared to those in such situations not reporting NDEs, the former group do not exhibit as much avoidance behavior. As such, they are not any more likely to develop post-traumatic stress disorder (Greyson, 2001). Similarly, post-traumatic growth seems more likely among those declaring NDEs, thereby suggesting that the NDE confers some protective influence (Khanna and Greyson, 2014; Khanna and Greyson, 2015). Such indications highlight the importance to gain a comprehensive understanding of the exact challenges faced by experiencers afterward.

## Sub-threshold NDEs

It is also worth mentioning that the majority of the published data on this topic comes from studies that focus on NDEs that were characterized using standardized scales such as the Greyson NDE scale (Greyson, 1983) or the Near-Death Experience Content (NDE-C) scale (Martial et al., 2020a). By definition, the transformative impacts in experiences that are not considered as typical NDEs, that is, with subjective experiences not considered as sufficiently rich as determined by the scales (i.e., not reaching the validated cut-off scores (Greyson, 1983; Martial et al., 2020a), which may be termed “sub-threshold”) remain unexplored. All these experiences, irrespective of their classification as “typical” NDEs by these scales, and the features therein, hold significance, especially from a clinical perspective. For instance, there may be challenges of psychological integration, as aforementioned, for patients, even if they were to report a single feature from the broader constellation which characterizes a “full” NDE. This is owing to the fact that each of these features are of an “extraordinary” nature in some respect, the most common of which encompassing the well-known phenomenon of out-of-body experience (OBE), a preternatural light, sense of transcendence of time, and encountering deceased loved ones (Charland-Verville et al., 2014). Such exceptional experiences, for instance in comparable psychedelic experiences, including the OBE, as later expanded, can lead to distressing ontological integration processes (Argyri et al., 2025). On the other hand, for many these features will be instrumental in beneficent effects, for example, other features have been specifically correlated with positive outcomes, such as encountering “mystical beings” or being shown a review of one’s life being associated with reduced fear of death (Pehlivanova et al., 2023). There is currently no empirical literature on the potential predictors of positive or negative enduring responses to having had an NDE. There are, however, at least some suggested hypotheses, supported by psychedelic literature, for predicting the quality of the acute experience, such as the mental and emotional context. A higher proportion of suicidal attempt contexts in distressing NDEs was observed by Cassol and colleagues (Cassol et al., 2019), suggesting a potential relationship between the negative emotional context of suicides and the resulting subjective experience.

## Differentiating from pathology

Another important point of clinical interest may be to differentiate NDEs from other episodes such as delirium, a common complication in critically ill patients (Stollings et al., 2021). Although empirical studies properly investigating the comparison of these two types of episodes are still needed, their respective subsequent impacts would certainly be very different. The occurrence of an NDE was recently delineated from (albeit not delirium *per se*) dream-like or delusional-type experiences in cardiac arrest wards via interview (Parnia et al., 2023), but NDEs can also be usefully identified to distinguish them from such delusions/hallucinations or delirium using the NDE-C scale (Martial et al., 2020a). Obviously, NDEs could occur in parallel to these more pathological states in single patients, as recently reported after an NDE from infection complication simultaneous with post-intensive care syndrome (Al Mutair et al., 2024). If an NDE (or indeed any other unusual subjective experience with some correspondence to the features on the NDE scales) vs. these other states is identified, and thus isolatable from them, then it would be advisable to recommend appropriate follow-up care unique to the needs of an NDE experiencer, and indeed of the specific experiencer (e.g., their belief systems). It would be important for such care to be done with a professional who is acquainted with the NDE, or at least will not seek to pathologize the experience. This is of course unless there are legitimate indications for such medicalization, for instance distressing psychotiform reactions to the NDE.

## Continued awareness of surroundings

In some publications, NDEs have been potentially involved with some continued connection to the surrounding environment (more specifically in the case of OBE), referring to episodes of connected consciousness (Martial et al., 2020b). Indeed, there are several lines of evidence, although not yet empirically confirmed by convincing methods (Martial et al., 2024a), suggesting that near-death experiences in otherwise apparently disconnected conditions still correctly report surrounding items and events (van Lommel et al., 2001; Greyson, 2003a; Sartori et al., 2006). While some strongly reference these to argue for a form of non-corporeal perception, they may only be perceiving a highly compelling simulated environment rather than the real environment (King, 2023). Further research with a sufficiently rigorous methodology to reliably confirm that these memories are really associated with real-life events is still needed, although an ongoing study is currently addressing this need (ClinicalTrials.gov registration: NCT06362525). The idea that patients may be aware of their surroundings, including their possibly critical state, in emergency contexts may be undesirable and naturally a concern for the emergency physicians. With the potentiality of such awareness, it is of critical importance that patient experience be taken into due consideration such that it can be improved. This may initially be as simple as care staff being cognizant of this eventuality and systematically behaving around, or toward, the patients accordingly, whether during or following the care.

## Psychological and neural identifying measures

The next step would be to incorporate a convenient means of identifying NDEs into care plans, which could be challenging today given emergency and ICU healthcare professionals’ time constraints. The simplest approach to garnering an initial idea of whether patients may have had an NDE is simply to gently ask them after regaining consciousness if they experienced anything unusual. Patients are unlikely to volunteer these spontaneously for several reasons, and even if directly inquiring, these same reasons may apply as to why they may not be readily forthcoming (e.g., personal embarrassment, having psychologically troubling experiences, fear of psychiatric intervention). Hence these inquiries should be prefaced by sharing with patients that these experiences are not necessarily pathological, and while priming should be minimized, very brief psychoeducation about NDEs could be possible here to foster comfort in reporting anything related. Even prior to asking patients, perhaps the most important practice would be to ensure the cultivation of trust, rapport and a safe space such that patients feel relaxed about being open to clinical staff about experiences which may have significant implications for their clinical trajectory. After identifying these experiences, to then home in on an accurate NDE assessment, the NDE-C scale should be employed.

Concurrently with retrospective, psychometric identification, we hold optimism for the development of innovative real-time brain monitoring technology that will ultimately enable the immediate identification of such experiences. Using brain markers as sources for automated detection of conscious experiences in critical conditions, without depending solely on memory, may ultimately assist medical management. This may be developed in the light of the recent interesting empirical studies suggesting surges of neurophysiological coherence and connectivity in the dying human brain, which has been speculated to be linked to the emergence of NDEs (Xu et al., 2023; Vicente et al., 2022; Mashour et al., 2024), following on from prior studies also showing high frequency, typically frontal, activity sometimes consistent with gamma (Auyong et al., 2010; Chawla et al., 2009; Chawla et al., 2017).

## Pharmacological intervention and its ethics

NDEs may be identified in these ways, however, it is important to note that not all patients close to death report them. We still do not know to what extent which pharmacological substances used in emergency or critical settings may elevate or reduce the likelihood of such an experience, which highlights that this is just one key area of important future research. However, one can still hypothesize that a higher proportion of patients (than the incidence typically reported) may experience an NDE but do not recall it, and this could theoretically be linked to the administration of drugs with powerful sedative and/or amnesic properties. Indeed, while NDEs may still occur under such conditions (Greyson et al., 2013) or even be elicited by some specific agents (for instance with dissociative properties), pharmacologically-induced comas with other agents, like propofol, would almost certainly lower the chance of having, or at least recalling, any NDE-type experience which may otherwise take place (Bonhomme et al., 2020)—and which may be of a profound nature with, potentially, enduring positive impacts on the person. This tension, of course, between minimizing patient distress from their medical symptoms by administering such drugs, while also not wishing to suppress any potentially personally salient experience will be one of important clinical judgment. However, some research does suggest that prolonged, deep sedation or such medically-induced comas may not only be over-relied upon, with alternative protocols being proposed and successfully implemented with some indications, but that their harms (e.g., neurocognitive) may sometimes outweigh their benefits (Pearce and Pearce, 2024). And while such pharmacological induction may suppress (usually positive) NDE-type memories as aforementioned, it may still possibly contribute to very distressing interior experiences which may be consistent with the “negative”-type NDEs discussed above (Pearce and Pearce, 2024).

One pertinent analog to this clinical dilemma is one espoused by Yaden and Griffiths (Yaden and Griffiths, 2020)—in relation to psychedelic substances, to be discussed further below—in debating whether psychiatrists should either prioritize actual psychedelics, or an emerging class of neuroplasticity-promoting drugs without the associated psychedelic subjective states, when treating patients. While the debate is ongoing, and legitimately so (see (Cheung et al., 2023)), Yaden and Griffiths (Yaden and Griffiths, 2020) resolutely endorse the former (even if this may risk provoking challenging experiences in some), fundamentally owing to psychedelics’ engendering of deeply meaningful mystical-type experiences, not unlike NDEs. “Challenging” here refers to experiences which may otherwise be termed “negative,” but with the understanding that such binary valuation may not be helpful, where for instance many such experiences would not ultimately be considered negative by those reporting them but instead sometimes lead to gratitude for the value found in the struggle (e.g. Argyri et al., 2025).

## NDEs as psychedelic-like experiences: predictors, context, and challenging experiences

Recent research on the modeling of NDEs with psychedelic substances has shown that, for instance, NDEs’ more general phenomenological features are replicated by DMT (Timmermann et al., 2018), their mystical dimensions such as sense of unity or ego dissolution are replicated by 5MeO-DMT (Michael et al., 2023), or the semantic properties of their narratives are replicated by monoaminergic psychedelics or especially dissociative anesthetics with psychedelic effects like ketamine (Martial et al., 2019; Martial et al., 2024b). Psychedelics are thus an example of “near-death-like experiences” (NDEs-like), i.e., sharing phenomenology in the absence of threat to life. Indeed, ketamine is routinely used in ICU settings, and so much of what is discussed herein in terms of identification and management of NDEs in such settings could be applicable to NDE-like episodes induced by ketamine administration (Corazza, 2008).

It is much beyond the scope of this article for an extensive review, but there is an increasingly expanding evidence base for psychedelics’ effectiveness as psychiatric treatments when in controlled settings (e.g., Schimmers et al., 2022; Ko et al., 2022). Although, non-clinical usage (albeit not *due* to being non-clinical) can sometimes lead to very distressing experiences (e.g., Johnstad, 2021) and extended difficulties, including of an existential nature, with psychiatric implications (e.g., Evans et al., 2023; Argyri et al., 2025). Relevantly, despite such personal substance use still entailing self-administration (and so anticipation of the altered state of consciousness), the uncontrolled settings and possibly lack of psychological preparedness (“set”) mirror the fact that NDE contexts involve their occurring to anyone upon an, often sudden, life-threatening event. In this way NDEs may represent a psychedelic-type experience without any controlled set and setting, which elevates the chance of challenging experiences. Also, similarly to NDEs, the exact determination of whether psychedelic experiences, or their outcomes, will be positive or challenging is complex. Some psychological traits including low openness or high neuroticism, or low surrender or disorganized psychological states are correlated with challenging experiences, while states of acceptance and surrender correlated with the opposite (Aday et al., 2021). Components of the setting are also crucial, where elements such as natural environments or the use of specific music are reported to be conducive to positive or de-escalate challenging experiences (McCartney et al., 2023).

These variables being considered, in light of NDEs’ psychedelic-like quality, NDEs may theoretically be similarly vulnerable to such contextual factors, which may have important clinical implications for improving patients’ experiences. Naturally, when considering their emergence in acute care settings, only so much can be achieved to optimize the patients’ psychological state, predominantly through paying attention to the settings of which they may gain awareness—such as staff’s generally empathetic approach to patients, communication during the acute event (e.g., cardiac arrest) both with the patient and between the doctors and nurses (e.g., striving to optimize a calm care-setting with clear and correct communication). The ability to beneficially modulate the setting may be further limited by a finding that baseline trait variables, such as anxiety, about which nothing may be done, conferred the most significant effects on changes in wellbeing associated with psychedelic experiences (Haijen et al., 2018). As well as this, though the above emphasizes the need for clinician attentiveness given psychedelic states’ and thus NDEs’ environmental sensitivity, those psychedelics which may actually be the most NDE-resembling like DMT or ketamine, are also the more dissociative, engendering new immersive, interior worlds, just as the NDE is considered a state of disconnected consciousness with a rich interiority (Martial et al., 2020b). As such, these psychedelics and NDEs may instead be less sensitive to the above “set and setting” effects, though this is speculation and further testing of this is indicated. Indeed, the fact that most NDEs are positive and one of the commonest features is a sense of peace, in spite of the critical condition or proximity to death of the individual, may support this idea (though, neurochemical reasons such as the release of endorphins may contribute to this, Martial et al., 2020b).

Despite all this, the OBE state is often autoscopic including impression of the surroundings, which regardless of the person’s interior experience again highlights the need for clinicians to be cognizant of the patient’s memories, which may manifest in the variety of manners delineated above, ranging from full NDEs to certain isolated features. Given at least some experiential link between NDEs and some psychedelics, we can lend from psychedelic-assisted therapy and recreational use in their curation of context to minimize harm and enhance therapeutic effects, such as the aforementioned natural environments or patients’ preferred music (McCartney et al., 2023).

## Population-specific care

Finally, there are still significant gaps in the NDE literature pertaining to their patent clinical pertinence. There is virtually nil on individuals with psychiatric diagnoses, including those in inpatient institutions, which is lamentable given interesting suggestions that, among those who had been close to death, those who also reported NDEs had less psychological distress versus those who did not report them, gesturing at protective influence of the NDE (Greyson, 2003b). Future research may need to expand its scope to ensure that specific groups, such as these, are not excluded from empirical studies for ethical concerns, especially given the burgeoning, ethical research on NDEs in other clinical populations. A greater ethical argument may be made, in fact, for the need to understand NDEs’ presentations in these vulnerable populations so that they can receive the best care, and to comprehend how NDEs’ may be protective or beneficial.

Additionally, other vulnerable populations such as children, who especially require attentive care in such acute contexts, have not been extensively studied in terms of their NDEs. Pediatric NDE incidence has been suggested to be as high as 58% (Thomas and O’Connor, 2024, or as high as 64% in a small sample Morse et al., 1986), markedly higher than the general population, which could be an overestimation and thus already indicating the need for further research. It appears that children report the same essential features of NDEs as adults (Morse et al., 1986), however with possible minor differences, including less frequent life reviews and encounters with the deceased, more animals (e.g., pets, International Association for Near-Death Studies, 2025). A less complex structure or less extensive features has been reported (Thomas and O’Connor, 2024), however others offer evidence that this may not be so (Long, 2014; Sutherland, 2009), where simpler language use may obscure more complex original experiences at least in qualitative studies. Purporting such similarity leads some to argue against a social constructivist case for NDE features, as the younger the children the less conditioned by expectation they may be—however, this is speculative, and the greater fantasy proneness, absorption or other adjacent psychological variables may make them even more suggestive to even minimal exposure to such themes. Enduring effects also appear to be comparable, but an accelerated maturation and attendant alterations in developmental trajectory may be occurring (International Association for Near-Death Studies, 2025), plausibly due to having intense mystical-type experiences at such tender ages—though, the plasticity of children’s brains could help to mitigate any challenging impacts. NDE valence also appears to include less distressing NDEs, at 3% versus 14% (Morse, 2013; Cassol et al., 2019). While such putative differences would likely be owing to children’s significantly different neural developmental stages and limited life experience, more research is certainly needed to substantiate current claims about childhood NDEs—especially where their identification and management in acute care settings may help ensure healthy development and/or buffer any challenges posed to it.

It should also be noted that while this piece focuses on those who actually report NDEs when near death, the subgroup of individuals who endure life-threatening experiences but do not report the altered state of the NDE should also be paid close clinical attention to given the likelihood of suffering trauma. In fact, some evidence suggests that having an NDE when near death may be partially protective against developing PTSD symptoms versus nearing death without reporting NDEs (Greyson, 2001; Khanna and Greyson, 2014; Khanna and Greyson, 2015).

## Conclusion

**Figure 1:**
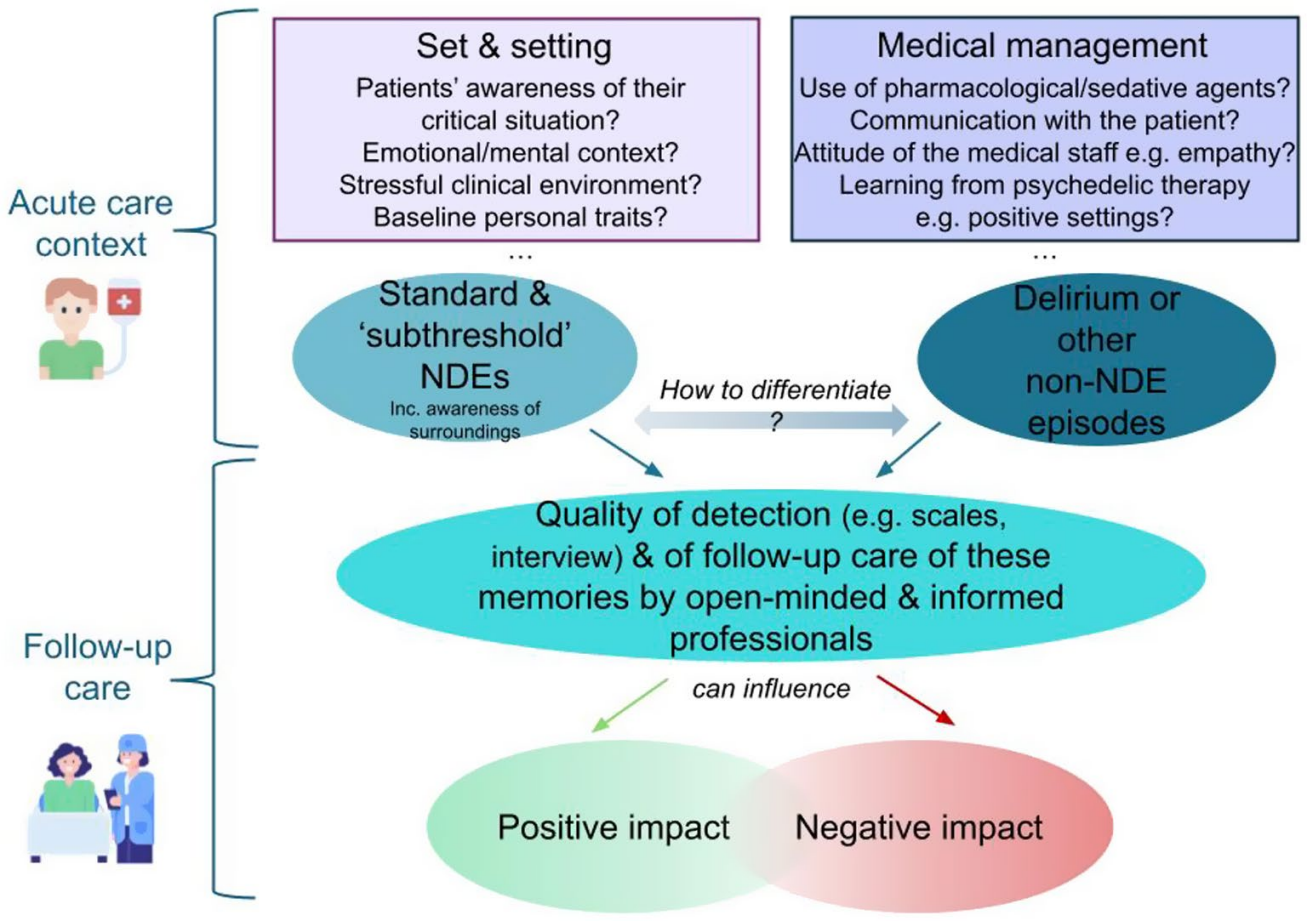
Acute: patients receiving emergency care may experience NDEs. Deep sedation should be reconsidered. Staff should ask patients if they experienced any altered state. NDEs must be distinguished from other altered states. Follow up: Given NDEs’ often entailing sustained awareness, and their sensitivity to psychological set and physical setting, patients must be given utmost care with this in mind, ultimately to minimize any negative outcome. N.b. “Subthreshold NDEs” refers to patient experiences which do not exceed the threshold on standard NDE scales, but which should still be included in the clinic and research.

In conclusion, we think that (prospective) NDE research holds heightened significance in the present day, particularly in acute care settings, which is precisely the classical context of occurrence of NDEs. Given the above-mentioned reasons, we stress the clinical significance of conducting systematic interviews with all post-emergency patients and ICU survivors to explore any potential memories upon awakening. In paying attention to NDEs and their features in acute care settings, a pathway for positive outcomes can be identified, important for both clinical care and possible data collection for further research. A graphical depiction of these conclusions can be found in Figure 1.

## Data Availability

The original contributions presented in the study are included in the article/supplementary material, further inquiries can be directed to the corresponding author.
